# Facile Hydrothermal Synthesis and Resistive Switching Mechanism of the α-Fe_2_O_3_ Memristor

**DOI:** 10.3390/molecules29235604

**Published:** 2024-11-27

**Authors:** Zhiqiang Yu, Qingcheng Wang, Jinhao Jia, Wenbo Kang, Meilian Ou, Zhimou Xu

**Affiliations:** 1 Faculty of Electronic Engineering, Guangxi University of Science and Technology, Liuzhou 545006, China; 221076997@stdmail.gxust.edu.cn (Q.W.); 221077051@stdmail.gxust.edu.cn (J.J.); 221077056@stdmail.gxust.edu.cn (W.K.); oumlian@mail2.sysu.edu.cn (M.O.); 2 Wuhan National Laboratory for Optoelectronics, School of Optical and Electronic Information, Huazhong University of Science and Technology, Wuhan 430074, China; xuzhimou@mail.hust.edu.cn

**Keywords:** hydrothermal method, α-Fe_2_O_3_ nanowire arrays, memristor, nonvolatile, conductive channels, oxygen vacancies

## Abstract

Among the transition metal oxides, hematite (α-Fe_2_O_3_) has been widely used in the preparation of memristors because of its excellent physical and chemical properties. In this paper, α-Fe_2_O_3_ nanowire arrays with a preferred orientation along the [110] direction were prepared by a facile hydrothermal method and annealing treatment on the FTO substrate, and then α-Fe_2_O_3_ nanowire array-based Au/α-Fe_2_O_3_/FTO memristors were obtained by plating the Au electrodes on the as-prepared α-Fe_2_O_3_ nanowire arrays. The as-prepared α-Fe_2_O_3_ nanowire array-based Au/α-Fe_2_O_3_/FTO memristors have demonstrated stable nonvolatile bipolar resistive switching behaviors with a high resistive switching ratio of about two orders of magnitude, good resistance retention (up to 10^3^ s), and ultralow set voltage (V_set_ = +2.63 V) and reset voltage (V_reset_ = −2 V). In addition, the space charge-limited conduction (SCLC) mechanism has been proposed to be in the high resistance state, and the formation and destruction of the conductive channels modulated by oxygen vacancies have been suggested to be responsible for the nonvolatile resistive switching behaviors of the Au/α-Fe_2_O_3_/FTO memristors. Our results show the potential of the Au/α-Fe_2_O_3_/FTO memristors in nonvolatile memory applications.

## 1. Introduction

In recent years, the development of semiconductor technology has promoted the progress of nanotechnology, and people’s requirements for the accuracy, stability, reliability, conversion speed and environmental protection of the device have gradually increased; The memristor, as a new micro-electronic device, is the fourth basic component of circuit engineering, alongside resistance, capacitance, and inductance. It can “remember” the amount of charge that runs through it, a property that allows the memristor to maintain its state after the power is turned off, unlike traditional resistors [1,2,3,4,5,6,7,8,9,10,11,12,13,14,15,16,17,18,19,20,21,22,23,24,25,26]. The memristor has received increasing attention because of its fast memory speed, high integration density, and multilevel properties, which means that it has a higher storage density, and can improve the speed of operations. The concept of the memristor was first proposed, along with mathematical proof, by Leon O. Chua in 1971 [2]. Currently, some companies including HP, SK Hynix, and others are developing and optimizing memristor technology. They have observed memristor phenomena in a variety of material systems and are trying to apply these materials to the next generation of nonvolatile memory. For example, HP LABS announced a TiO_2_-based RRAM (impedance memory) device in 2008, which was the first time a physical model of a memristor had been constructed, proving the existence of a memristor device, and serving as an important milestone in the development of memristor technology [3].

The memristor is usually a “sandwich” structure, and its components are generally the upper and lower two electrodes and a resistive layer. At present, there are many kinds of resistive layer materials that have been discovered and applied in practice. Researchers try to improve the performance of the memristor by combining a variety of materials and designing structures. Among the discovered resistive materials, one-dimensional nanomaterials, especially the new nanomaterials represented by semiconductors and metal oxide nanowire (nanorods), have important research value in the fields of nanodevices and optoelectronic devices due to their special physical and chemical properties. Nanowires (nanorods), as the building unit of a new type of optoelectronic device, can be combined with composite functional units by layer-by-layer assembly and nano connection to produce micro-devices with new functions. Nanowire (nanorods) can promote the transport of charge carriers, and their introduction into the memristor can improve the memory performance of the device. The transition metal oxide nanomaterials can change the conductive channels by applying an electric field, promoting charge and carrier migration in their interior, and then causing a great change in resistance. Specifically, when a voltage is applied to a memristor device, a narrow “conductive channel” is created in the oxide material, through which a current can flow inside the material. When the applied voltage is removed, the conductive channel closes again, returning to the initial high-impedance state. In addition, metal oxide nanowire arrays can be grown on different substrates by hydrothermal methods, providing multiple possibilities for the design of memristor in nonvolatile memory applications.

With the improvement in people’s requirements for device accuracy, stability, reliability, conversion speed, environmental protection, etc., traditional materials have gradually been unable to meet people’s needs, which has prompted researchers to develop several memristor materials that not only have excellent electrical and thermal properties, but also provide higher stability and reliability. To date, researchers have found a variety of oxide materials with resistive switching properties, such as Al_2_O_3_, Cu_x_O [4,21], TiO_2_ [5,8,9], Fe_2_O_3_ [20], and ZnO [6,7,19], which often act as dielectrics in memristors. Among them, the hematite has become an excellent candidate material for the construction of memristors due to its narrow band gap width (2.1~2.2eV), excellent photoelectric performance, chemical stability, non-toxicity, low cost and abundant storage [10]. In 2015, Yan Xiaobing et al. [11] of Hebei University used ultrasonic spray pyrolysis to prepare an α-Fe_2_O_3_ nano-film memristor on FTO conductive glass. The test results showed that the device had bipolar resistive switching characteristics and good stability, but the resistive switching ratio of the device could only reach about one order of magnitude. In 2023, Muhammad Tahir et al., Department of Physics, Faisalabad University, Pakistan, synthesized hematite (α-Fe_2_O_3_) nanoparticles by the co-precipitation method and the sol–gel method [12]. The structures of α-Fe_2_O_3_ nanoparticles were characterized by X-ray diffraction (XRD) and analyzed by scanning electron microscopy (SEM). The crystalline sizes of the samples prepared by the two methods were determined by using the Scherer formula. It was found that the sample prepared by the coprecipitation method was 12% smaller than that prepared by the sol–gel method. The test results showed that the phase transition from goethite to hematite changed the band gap and structure. The thermal effect of the iron oxide nanoparticles showed that the band gap was reduced, but the resistive switching ratio of the device was still low. In 2024, N. Peavithra et al. [22] from India, studied the effect of pH change during the synthesis of α-Fe_2_O_3_ nanoparticles and Ni-doped α-Fe_2_O_3_ nanoparticles. The α-Fe_2_O_3_ and Ni-doped α-Fe_2_O_3_ nanoparticles with pH variations (5, 7, and 12) were synthesized by the co-precipitation method using Vinca extract. During the synthesis of the nanoparticles, the effects of pH on their structural properties, morphology, band gap, magnetic properties and antibacterial properties were observed. The synthesized nanoparticles had a rhomboid hematite structure, and the EDS spectra confirmed the peaks corresponding to Fe, Ni, and O, while the XRD pattern indicated the formation of a hematite phase. FTIR spectroscopy confirmed the existence of chemical bonds in the α-Fe_2_O_3_ and Ni-doped α-Fe_2_O_3_ nanoparticles. The VSM results confirmed that the Ni-doped α-Fe_2_O_3_ prepared at pH = 5 and 12 exhibits weaker ferromagnetic behavior than the samples prepared under pH7, possibly due to the morphology effect of the nanoparticles. In current studies, many researchers are exploring different metal/oxide/substrate structures to improve photoelectrochemical properties. For example, in 2020, Ren Zhijun et al. [23] have designed an Ag/α-Fe_2_O_3_/Ti memristor and compared the typical bipolar resistance storage behavior of the device under dark and light conditions, compared with the resistance ratio increased by several orders of magnitude under white light conditions. However, compared with the above literature, the α-Fe_2_O_3_ nanowire array-based Au/α-Fe_2_O_3_/FTO memristors in this paper play a role in enhancing light absorption and charge separation by introducing Au electrodes. The interface modification between the α-Fe_2_O_3_ nanowire arrays and the FTO substrate optimizes the electron transport path and reduces the charge recombination at the interface.

In the preparation processes of the α-Fe_2_O_3_ nanowire array-based Au/α-Fe_2_O_3_/FTO memristors, the hydrothermal method is low cost compared with the above-mentioned ultrasonic spray pyrolysis method, co-precipitation method, and sol–gel method; it can reduce the production cost, has good uniformity, high crystallinity, and controllable morphology, and can be used for the synthesis of large-scale nanomaterials. It has obvious advantages in ensuring material quality, improving production efficiency, and reducing costs. The memristor prepared in this paper uses the hydrothermal method and annealing process to grow a layer of dense α-Fe_2_O_3_ nanowire arrays on the FTO conductive glass, and then a layer of metal electrode is plated to the α-Fe_2_O_3_ nanowire arrays by the magnetron sputtering method. The Au electrode used in this paper has a thickness of 100nm. Finally, the Au/α-Fe_2_O_3_/FTO memristors are designed. FTO (fluorine-doped titanium oxide) substrate is chosen because it has a high conductivity and can provide good current transmission performance, which is conducive to the movement of charge carriers and signal transmission in the memristor. It has good chemical stability and is not susceptible to environmental factors when preparing nano-devices by the hydrothermal method, which helps to maintain the stability and reliability of the memristor. With proper work function, it can form a good interface with metal oxides, which helps to improve the switching performance and stability of the memristor. The Au/α-Fe_2_O_3_/FTO memristors designed by the hydrothermal method not only save cost, but also obtain denser α-Fe_2_O_3_ nanowire arrays; the resistance switching ratio is also improved, as well as the stability of the membrane devices. Therefore, this paper chose the hydrothermal method to design the Au/α-Fe_2_O_3_/FTO memristors. It is concerned with summarizing the experimental phenomena, paying attention to purity, selecting suitable preparation methods, optimizing the reaction conditions, conducting material characterization and analysis, analyzing the mechanism and discovering the involved physical laws, further analyzing its resistance switching mechanism, and then exploring the performance optimization and application expansion of α-Fe_2_O_3_. The design of a low cost, high density nonvolatile Au/α-Fe_2_O_3_/FTO memristors have important significance for the future nonvolatile memory applications.

## 2. Experimental

### 2.1. Experimental Materials

The fluorine-doped tin oxide-coated glass (FTO, 15 Ω/square) used as the bottom electrode of the α-Fe_2_O_3_ nanowire array-based Au/α-Fe_2_O_3_/FTO memristor was purchased from the NSG Pilkington (Tokyo, Japan), and the ferric nitrate nine-water (Fe(NO_3_)_3_·9H_2_O, 99%) was purchased from Aladdin Company (Fukuoka, Japan). The chemical reagents used in the experiments were all analytically pure, and no further purification was required before use. The experimental equipment used in this article included an X-ray diffraction (XRD, PANalytic PW3040/60, Malvern, UK), the scanning electron microscopy (SEM, Nova NanoSEM 450, FEI, Hillsboro, OR, USA), the X-ray photoelectron spectroscopy (XPS, AXIS-ULTRA DLD-600W, Shimadzu, Kyoto, Japan) and an Agilent B2901A source meter (Santa Clara, CA, USA).

### 2.2. Experimental Process

First, the experimental equipment consists of the inner tank, beaker, magneton, tweezers, medicine spoon, and pipette gun. To clean the residual trace impurities in the inner liner, a high-power ultrasonic machine was used for cleaning, and then the FTO substrate was cleaned. The cleaning solution was mixed with anhydrous alcohol, isopropyl alcohol, and acetone in a ratio of 1:1:1, and then the FTO substrate was put into the cleaning solution for ultrasonic cleaning and repeated, for 15 min each time; the purpose was to clean the organic matter adhered to the FTO substrate. Then, it was changed to pure water and alcohol in the ratio of 1:1, mixed, ultrasounded twice, and finally cleaned only with alcohol and ultrasounded twice; the purpose being to clean the dust that may have been adsorbed by the FTO substrate or any impurities existing in the beaker to avoid affecting the experimental results. After cleaning, the two FTO substrates were taken out and rinsed once with pure water and alcohol, and then put in a petri dish with a layer of filter paper to dry naturally. The remaining FTO substrate was placed in a beaker filled with alcohol, covered with plastic wrap, and sealed for future applications.

**Figure 1:**
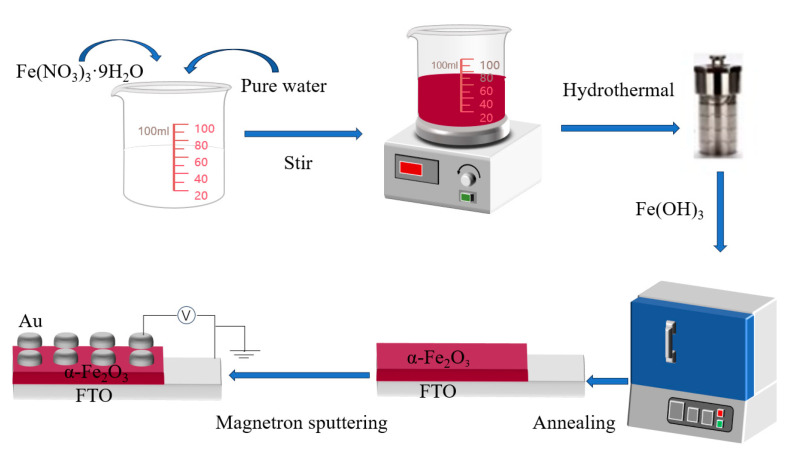
Preparation process of the Au/α-Fe_2_O_3_/FTO memristor.

**Figure 2:**
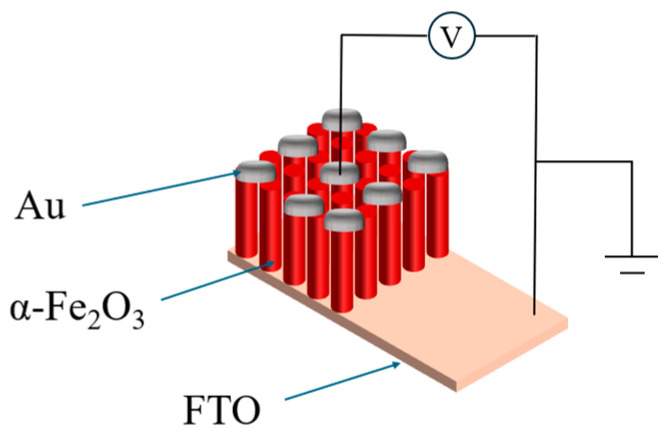
Schematic model of the Au/α-Fe_2_O_3_/FTO memristor.

The α-Fe_2_O_3_ nanowire arrays were prepared by a facile hydrothermal method and annealing treatment on the FTO substrate, and the filling ratio of the reactor is about 80%. First, the α-Fe_2_O_3_ seed layer was prepared, and the precursor solution was made by mixing ferric nitrate nine-water with pure water. The concentration of precursor solution configured in the experiment was 0.05 mmol@15 mL, 0.1 mmol@15 mL, 0.25 mmol@15 mL, 0.5 mmol@15 mL, 0.75 mmol@15 mL, and 1 mmol@15 mL, respectively. The thickness of the required nanofilms could be obtained by changing the concentration of the solution. The experimental results showed that when the concentration of precursor solution is within a certain range, the thickness of the α-Fe_2_O_3_ nanowire arrays prepared by the hydrothermal method gradually becomes thicker with the increase of concentration. In this study, when the concentration of precursor solution changed from 0.05 mmol to 1 mmol, the thickness of the α-Fe_2_O_3_ nanowire arrays gradually become thicker. The prepared mixed solution was placed on a magnetic stirrer, the rotational speed was set at 600 rpm/min, and the liquid was stirred at room temperature for 15 min to obtain a brown and transparent precursor solution. Meanwhile, the temperature and humidity conditions of the experiment were recorded. The conductive side of the dried FTO substrate was tilted downward into the inner tank, and then 15 mL of the prepared precursor solution was added to the inner tank with a pipette gun. After the inner tank was placed into the reactor, it was sealed and heated in a Muffle furnace to 90 °C and kept at 90 °C for 2 h. After the hydrothermal reaction was completed, the reactor was taken out of the Muffle furnace and cooled to room temperature. After cooling, the sample is removed and then soaked in a mixture of pure water and anhydrous alcohol for one day, followed by anhydrous alcohol for another day. The purpose of soaking was to remove the impurities chemisorbed on the surface of the sample. The Fe(OH)_3_ layer can grow directly on the conductive surface of the FTO substrate with the hydrothermal method, but there is still some residual trace impurities chemisorbed on the non-conductive surface of the FTO substrate after the hydrothermal process, which can be removed by soaking. After the above process was completed, the conductive side of the dried sample was sealed in the Muffle furnace, and the dense α-Fe_2_O_3_ nanowire arrays were obtained by annealing at 800 °C for 20 min. Then, a layer of Au with a thickness of 100 nm was plated on the surface of the α-Fe_2_O_3_ nanowire arrays sample by magnetron sputtering technology as the upper metal electrode. The Au layer deposition plays the role of the upper electrode in the final device structure, which is the channel for current entering and leaving the memristor device. It transmits the signal of the external circuit to the memristor layer and collects the signal from the memristor layer to realize the control of the device and the state reading from the device. A good contact interface was formed between the upper electrode and the memristor layer, which can lower the contact resistance, and reduce the energy consumption. Finally, the Au/α-Fe_2_O_3_/FTO memristors were obtained, and the corresponding preparation process and device model have been given in Figure 1 and Figure 2, respectively.

The crystal structure of the prepared samples was analyzed by X-ray diffractometry (XRD). The structure and appearance of the product were analyzed by field emission scanning electron microscopy (FESEM). The elemental composition and chemical state of the α-Fe_2_O_3_ nanowire arrays were analyzed by X-ray photoelectron spectrometry (XPS). The electrical properties of Au/α-Fe_2_O_3_/FTO memristors were measured with an Agilent B2901A equipment under normal temperature and pressure. During the test process, the voltage was applied from the upper electrode, Au, of the Au/α-Fe_2_O_3_/FTO memristors, and the resistance change of the device was tested by grounding the bottom electrode.

## 3. Performance Analysis

### 3.1. Physical Characterization

The hydrothermal synthesis can be carried out at the low temperature of 90 °C for 2 h, which makes the diffusion rate of the reaction material slow and conducive to the uniform deposition of nanoparticles on the substrate surface. The prepared α-Fe_2_O_3_ nanowire arrays show relatively uniform nanostructures on the surface and cross section images of FESEM, without obvious aggregation or vacancy phenomenon. The size and morphology of the nanoparticles can be controlled by adjusting the reaction conditions, such as temperature, reaction time, reactant concentration, etc. At lower temperature and atmospheric pressure conditions, the solubility of the reactive substance is higher, which is conducive to uniform nucleation and growth of nanoparticles, to achieve a higher nanomembrane material density. The α-Fe_2_O_3_ nanowire arrays prepared by the hydrothermal method show a relatively flat surface on the surface image of FESEM, without any obvious particle accumulation and uneven phenomenon. This flat surface is conducive to the preparation and performance improvement of subsequent devices.

**Figure 3:**
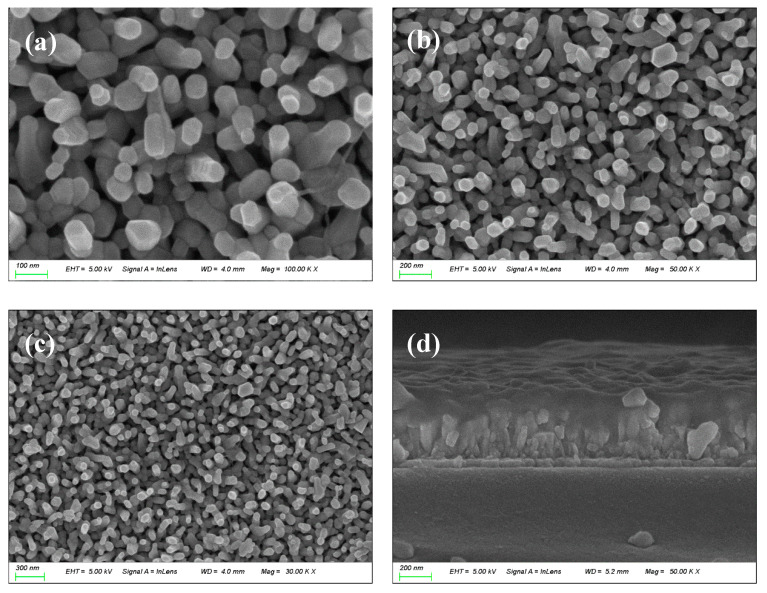
(**a**–**c**) Surface topographies under different magnifications, (**d**) cross-sectional topography of the as-prepared α-Fe_2_O_3_ nanowire arrays.

**Figure 4:**
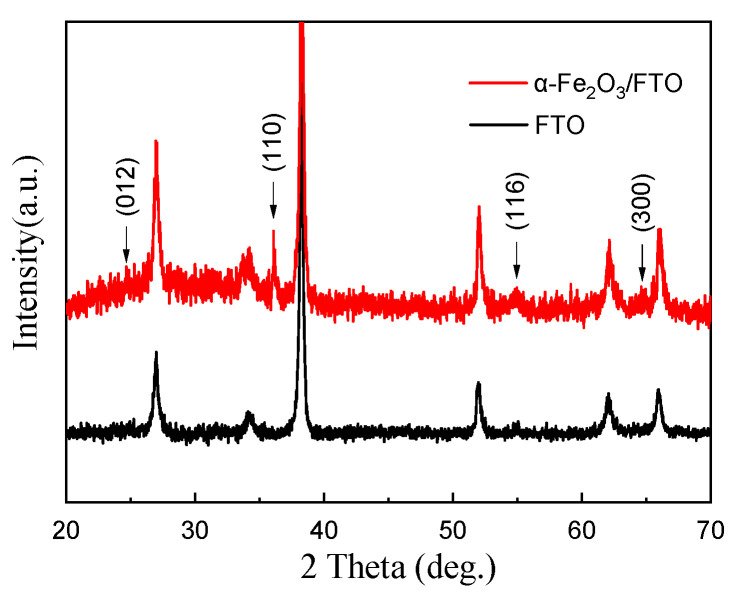
XRD pattern of the as-prepared α-Fe_2_O_3_ sample.

Figure 3a–c shows the FESEM images on the surface of the resistive material layer of the α-Fe_2_O_3_ nanowire arrays sample grown on the FTO conductive glass. From the surface topography, it can be seen that a layer of rod-like material grows on the FTO conductive glass, and the rod-like structure is randomly distributed and uniformly covered on the FTO substrate, forming a complex nanostructure. From the surface topography, it can be found that the α-Fe_2_O_3_ nanowire arrays prepared by the hydrothermal synthesis process are very dense, which is one of the advantages of the preparation of α-Fe_2_O_3_ nanowire arrays by the hydrothermal synthesis process. Figure 3d shows the FESEM image of the cross-section of the resistive material layer of the α-Fe_2_O_3_ nanowire arrays sample. The cross-section morphology of the α-Fe_2_O_3_ nanowire arrays were characterized by SEM, and the thickness of the resistive material layer was about 280 nm. Figure 4 shows the XRD pattern of the as-prepared α-Fe_2_O_3_ sample. To determine the structure of the prepared functional layer, the XRD pattern of the α-Fe_2_O_3_/FTO sample was tested in this paper. The above figure shows the pattern after deducting the FTO background. All the XRD diffraction peaks appearing on the as-prepared α-Fe_2_O_3_ sample can be classified as the rhombohedral phase α-Fe_2_O_3_ (PDF# 33-0664, JCPDS) [13,24]. In particular, the significantly enhanced (110) plane diffraction peak near 35.6° demonstrates that the as-prepared α-Fe_2_O_3_ sample are highly oriented with a preferred growth orientation along the [110] direction with respect to the FTO substrate.

**Figure 5:**
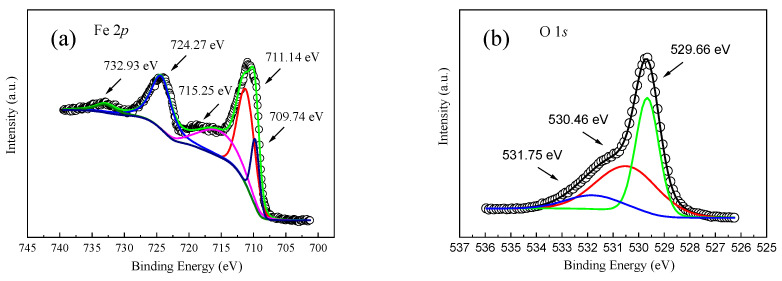
XPS spectra of the α-Fe_2_O_3_ nanowire arrays: (**a**) Fe 2p, (**b**) O 1s.

X-ray photoelectron spectroscopy (XPS) can provide information about the chemical composition of the surface of a sample at a depth of several nanometers. By analyzing the kinetic energy of the photoelectrons, the types and relative contents of each element on the surface of the sample can be determined. For α-Fe_2_O_3_ nanowire arrays, XPS detects iron (Fe), oxygen (O), and possible impurities. XPS can identify elements and can determine the chemical state of these elements by analyzing the binding energy. Different chemical states lead to slight changes in the binding energy of photoelectrons. The main purpose of XPS measurement in this paper was to determine the surface structure and chemical state of α-Fe_2_O_3_ nanowire arrays deposited on the FTO substrate [14]. Figure 5a shows an XPS diagram of the Fe 2p element in the sample, from which five peaks can be observed at 732.93 eV, 724.27 eV, 715.25 eV, 711.14 eV, and 709.74 eV. From the XPS peak spectrum corresponding to the Fe 2p orbit, it can be found that Fe 2p_3/2_ has a peak value at 711.14 eV, Fe 2p_1/2_ has a peak value at 724.27 eV, and the difference between the two peaks is 13.11 eV. The difference is the splitting energy of the Fe 2p orbit, which comes from the contribution of the metal–oxygen (Fe-O) bond. The Fe 2p XPS peak is correlated with Fe^2+^ at 709.74 eV [24]. In addition, two distinguishable satellite peaks can be observed in the figure, respectively located at 715.25 eV and 732.93 eV, indicating the presence of Fe^3+^ in the α-Fe_2_O_3_ nanowire arrays [4]. There are three peaks in the XPS peak spectrum corresponding to the O 1s orbital as displayed in Figure 5b, which correspond to the lattice oxygen, oxygen vacancy, and chemisorbed oxygen at the positions of binding energy of 529.66 eV, 530.46 eV, and 531.75 eV, respectively. The fitting peak at the binding energy of 529.66 eV corresponds to the metal–oxygen (Fe-O) bond, and the fitting peak at the binding energy of 530.46 eV corresponds to the oxygen vacancy (Fe-O-C) [15]. The chemical state of Fe was determined by XPS analysis. Through the analysis of XRD and XPS patterns, it can be found that there are still crystal defects in the Au/α-Fe_2_O_3_/FTO memristors prepared by magnetron sputtering, but it is precisely because of these crystal defects that transport channels for oxygen vacancies and electrons are provided. There are many oxygen vacancy defects in the prepared α-Fe_2_O_3_ nanowire arrays, forming a series of defect states. These vacancies can be used as the capture center, which can realize the controllable regulation of the device impedance performance and will affect the nonvolatile resistance switch switching behavior of the device. At the same time, due to the existence of oxygen vacancy, under the action of the electric field, the oxygen vacancy will move towards the cathode, resulting in a local oxygen-deficient region, resulting in the reduction of the chemical valence of the non-oxygen elements in the oxide, resulting in the generation of the Fe^2+^.

### 3.2. Electrochemical Characterization

In the test and control of the device, in order to avoid damage to the device, the limited current is set to 0.01 A, the bias voltage is applied to the Au electrode of the device, and the substrate FTO is always grounded during the test and control process. In our experiment, the DC measurement technique was used to measure the *I-V* characteristics.

**Figure 6:**
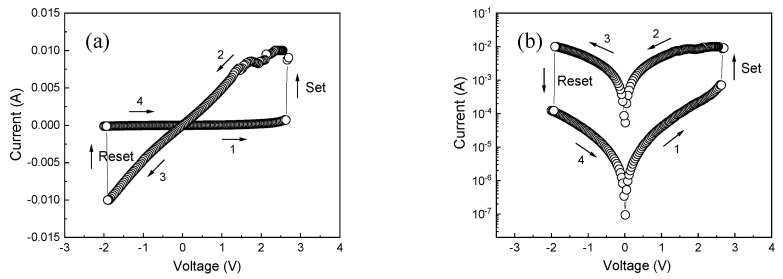
*I-V* characteristics of the Au/α-Fe_2_O_3_/FTO memristor: (**a**) The linear *I-V* curve of the device, (**b**) The semi-logarithmic *I-V* curve of the device.

To illustrate the resistive switching characteristics of Au/α-Fe_2_O_3_/FTO memristors, Figure 6a,b indicates the linear and semi-logarithmic current–voltage (*I-V*) curves of the as-prepared Au/α-Fe_2_O_3_/FTO memristors. The cyclic voltage scanning direction is 0 V → +3 V → 0 V → −2 V → 0 V. The arrows in the figure indicate the direction of the cyclic voltage scan, and the number indicates the scanning sequence. During the test, the external bias voltage has been applied to the top Au electrode, and the FTO substrate is always grounded. The Au/α-Fe_2_O_3_/FTO memristors are in a high resistance state at the beginning. As can be seen from the figure, when a gradually increasing forward bias voltage is applied from 0 V to +2.63 V, the resistance state is gradually changed from HRS to LRS, and then maintained at the LRS state, while the corresponding threshold voltage is +2.63 V. At this time, the device has a setting process, called the “set” process. The corresponding threshold transition voltage is called the set voltage (V_set_). When the negative bias voltage is gradually applied from 0 V to −2 V, the resistance state gradually changes from the LRS to HRS, and then remains in the HRS state; at this time the device has a reset process, the corresponding threshold voltage is −2 V, called the reset voltage (V_reset_), and the setting process and the reset process occur at opposite bias polarity. The results demonstrate the bipolar resistive switching behaviors of the Au/α-Fe_2_O_3_/FTO memristors.

As presented in Table 1, the as-prepared Au/α-Fe_2_O_3_/FTO memristors in this paper demonstrated nonvolatile resistive switching behavior with a relatively low set voltage (V_set_ = +2.63 V) and reset voltage (V_reset_ = −2 V), high resistance ratio (R_HRS_/R_LRS_) of more than two orders of magnitude, good resistance retention (up to 10^3^ s), and good durability compared with the previous literature results [10,11,13,14,18,25,26].

**Figure 7:**
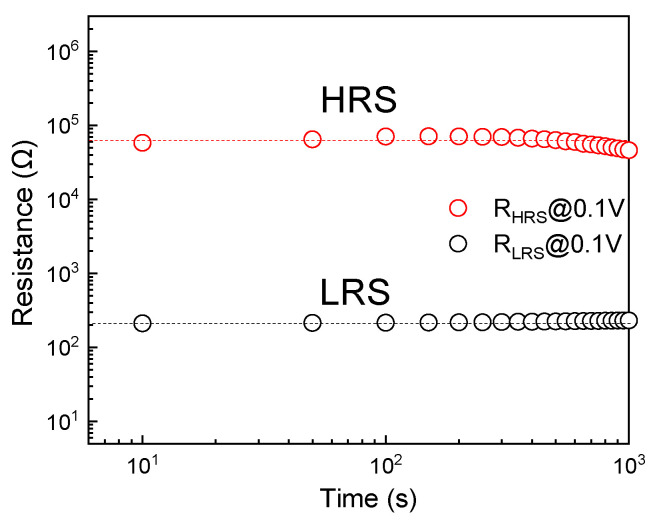
Retention characteristics of the device at the read voltage of 0.1V.

Figure 7 shows the test curve of time retention characteristics of the Au/α-Fe_2_O_3_/FTO memristors at a constant reading voltage of 0.1 V. Different voltages are applied to the device so that the α-Fe_2_O_3_ film reaches a certain resistance state, and then the resistance value is read using a voltage below 0.1 V, while the reading voltage is kept constant during the test. It can be seen from the figure that the ratio of the resistance value of the high resistance state to the low resistance state of the device is about 100, and it remains basically unchanged after 10^3^ s. The test results show that the Au/α-Fe_2_O_3_/FTO memristor is stable and has good stability and continuity.

**Figure 8:**
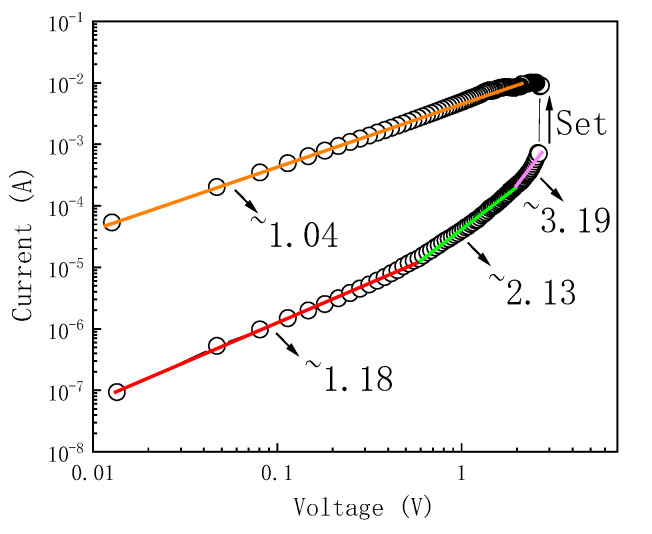
Double-logarithmic *I-V* fitting curve of the device in the forward bias voltage.

In order to study the resistance transition mechanism of Au/α-Fe_2_O_3_/FTO nano-memristors, the *I-V* curve in the positive voltage region was converted into double-log coordinates by using the log-form *I-V* curve to obtain the double-log fitting diagram of the high resistance state (HRS) and low resistance state (LRS) under the forward bias voltage as shown in Figure 8. The mechanism of resistive switching of the memristor is studied by piecewise fitting the log-log curves of the high and low resistance states. The fitting results show that the double-log curve of the low resistance state is a straight line with a slope of 1.04, indicating that the voltage is proportional to the current in this state; that is to say, the device mainly obeys Ohm’s law in this state, and the form of Ohm’s law is shown as follows Equation (1) [16].

(1) $$J_{ohm}=qn_{0}\mu E$$

where *q* is the basic charge, *n*_0_ is the density of the heat-generated free carrier, *μ* is the mobility of the carrier, *E* is the electric field strength, and *J* is the current density. Through the analysis of the low resistance state of the device, conductive filaments may be formed in the α-Fe_2_O_3_ nanowire arrays [17]. The double-log fitting curve of the high configuration also shows a straight line in the low voltage part with a slope of 1.18, which conforms to the ohmic conduction characteristics. With the gradual increase of voltage, the voltage and current of the nanodevices show a quadratic relationship, that is, $I\propto V^{2}$. When the voltage increases to a certain value, it enters the current surge stage, and the current and voltage increase exponentially, which meets $I\propto V^{n}$. From the above analysis, the Au/α-Fe_2_O_3_/FTO memristor satisfies the space charge-limited conduction (SCLC) mechanism. The SCLC mechanism can affect the resistive switching behavior of the device. At high voltage, the number of injected carriers (such as electrons) increases significantly, forming space charge-limited conduction, resulting in low resistance of the material. Under low voltage or reverse voltage, the injected carrier is reduced, the space charge effect is weakened, and the material returns to the high resistance state. The SCLC mechanism usually exhibits a nonlinear current-voltage (*I-V*) relationship. In a certain voltage range, the current increases exponentially with the increase of voltage, which is due to the enhancement of the space charge limitation effect. This nonlinear property can distinguish between different resistance states. At high voltage, due to the SCLC mechanism, the thermal effect and strong electric field effect caused by carrier injection will further affect the resistance switch behavior. The thermal effect may lead to a local temperature increase, further enhancing carrier mobility and improving conductivity. The strong electric field effect can promote the migration of oxygen vacancy and change the defective state of the material, thus affecting the resistance state. Oxygen vacancy defects introduce extra carriers (electrons or holes) into the resistive layer material, which can reduce the resistance of the material and change the conductivity and impedance characteristics of the resistive layer. Lattice deformation caused by oxygen vacancies can affect the mobility of electrons, increase the conductivity, and lead to changes in local electric fields, thus affecting the overall impedance characteristics. Oxygen vacancy defects can migrate under the action of an electric field, forming or destroying conductive channels: When a forward voltage is applied, the oxygen vacancy may migrate and accumulate, forming a low-resistance conductive channel, thereby leaving the device in a low-resistance state. When a reverse voltage or a larger forward voltage is applied, these conductive channels may be destroyed or reorganized, returning the device to a high resistance state. The formation and destruction of the conductive channels caused by the oxygen vacancies give the device bistable characteristics. Due to the stability of the oxygen vacancy, once the conductive channels are formed or destroyed, the device can maintain its conductive state even if the voltage is removed. This allows the memory states to be maintained for a long time, reflecting nonvolatility in future memory applications.

## 4. Conclusions

In summary, we successfully prepared an α-Fe_2_O_3_ nanomaterial resistance layer growing preferentially along the [110] direction on the FTO substrate through the hydrothermal synthesis process. The hydrothermal synthesis process reduces the production cost, and the prepared device has good uniformity, high crystallinity and controllable morphology, which can be used for the synthesis of large-scale nanomaterials. By analyzing the *I-V* curve, it was confirmed that the device has the characteristics of bipolar resistive switching. The resistive switching ratio of the device is about two orders of magnitude. The stability is good and can maintain more than 10^3^ s without significant changes. The space charge-limited conduction mechanism of Au/α-Fe_2_O_3_/FTO memristors was determined by the analysis of the log-log fitting curve. The effect of oxygen vacancy defects on the impedance of Au/α-Fe_2_O_3_/FTO memristors and the effect of the space charge-limited conduction mechanism on the resistance switching behavior of Au/α-Fe_2_O_3_/FTO memristors were studied. To summarize, this work provides a new idea for the preparation of the high efficiency and energy saving memristor devices.

## Figures and Tables

**Table 1:** Comparison of the parameters of the Fe_2_O_3_-based memory devices.

Device Structure	V_set_/V_reset_ (V)	Preparation Method	R_HRS_/R_LRS_ Ratio	Retention	Reference
Ag/Fe_2_O_3_/FTO	~+2/~−2	Hydrothermal method	~10^4^	-	[10]
Au/Fe_2_O_3_/FTO	~+1.5/~−1.2	Ultrasonic spray pyrolysis	~10	10 h	[11]
Ag/Fe_2_O_3_/ZnO/ITO	+0.9/−1	Spin coating technique	~90.1	30 days	[13]
Ag/Fe_2_O_3_-PVA/FTO	+2/−0.7	Co-precipitation method	~10	-	[14]
Ag/[BiFeO_3_/γ-Fe_2_O_3_]/FTO	+0.98/−1.38	Magnetron sputtering	~10	-	[18]
Ag/γ-Fe_2_O_3_ films/FTO	+1.85/−1.25	Spin coating technique	-	-	[25]
Ag/[TiO_2_/α-Fe_2_O_3_]/FTO	~+4/~−4	Hydrothermal method	~10	10^3^ s	[26]
Au/α-Fe_2_O_3_/FTO	+2.63/−2	Hydrothermal method	>10^2^	>10^3^ s	This work

## Data Availability

The data presented in this study are available on request from the corresponding author.
